# Demonstration of epitaxial growth of strain-relaxed GaN films on graphene/SiC substrates for long wavelength light-emitting diodes

**DOI:** 10.1038/s41377-021-00560-3

**Published:** 2021-06-03

**Authors:** Ye Yu, Tao Wang, Xiufang Chen, Lidong Zhang, Yang Wang, Yunfei Niu, Jiaqi Yu, Haotian Ma, Xiaomeng Li, Fang Liu, Gaoqiang Deng, Zhifeng Shi, Baolin Zhang, Xinqiang Wang, Yuantao Zhang

**Affiliations:** 1 State Key Laboratory of Integrated Optoelectronics, College of Electronic Science and Engineering, Jilin University, Qianjin Street 2699, Changchun, 130012 China; 2 Electron Microscopy Laboratory, School of Physics, Peking University, Beijing, 100871 China; 3 State Key Laboratory of Crystal Materials, Shandong University, Jinan, 250100 China; 4 State Key Laboratory for Mesoscopic Physics and Frontiers Science Center for Nano-optoelectronics, School of Physics, Peking University, Beijing, 100871 China; 5 Key Laboratory of Materials Physics of Ministry of Education, School of Physics and Microelectronics, Zhengzhou University, Zhengzhou, 450052 China

**Keywords:** Inorganic LEDs, Optical properties and devices

## Abstract

Strain modulation is crucial for heteroepitaxy such as GaN on foreign substrates. Here, the epitaxy of strain-relaxed GaN films on graphene/SiC substrates by metal-organic chemical vapor deposition is demonstrated. Graphene was directly prepared on SiC substrates by thermal decomposition. Its pre-treatment with nitrogen-plasma can introduce C–N dangling bonds, which provides nucleation sites for subsequent epitaxial growth. The scanning transmission electron microscopy measurements confirm that part of graphene surface was etched by nitrogen-plasma. We study the growth behavior on different areas of graphene surface after pre-treatment, and propose a growth model to explain the epitaxial growth mechanism of GaN films on graphene. Significantly, graphene is found to be effective to reduce the biaxial stress in GaN films and the strain relaxation improves indium-atom incorporation in InGaN/GaN multiple quantum wells (MQWs) active region, which results in the obvious red-shift of light-emitting wavelength of InGaN/GaN MQWs. This work opens up a new way for the fabrication of GaN-based long wavelength light-emitting diodes.

## Introduction

Due to direct wide bandgap and high stability, group III-nitride semiconductors have been widely used in light-emitting diodes (LEDs), laser diodes and photodetectors^1–3^. Currently, the high cost of native substrates makes group III-nitrides usually grow on foreign substrates, such as sapphire, SiC, and Si^4–6^. However, there are lattice and thermal mismatches between nitride films and foreign substrates, leading to the formation of high-density point defects, threading dislocations, and residual stress in the prepared films and devices, which degrade the working lifetime and efficiency of GaN-based optoelectronic devices^7–9^.

Recently, GaN films grown on graphene and other two-dimensional materials have attracted extensive attention^10–13^. The epitaxial growth of GaN on graphene forms weak covalent bonds at the interface between graphene and epitaxial layer, which makes the lattice at the interface produce no large strain as the traditional heteroepitaxy, and the stress in films greatly reduced^14^. This provides a new path for heteroepitaxial growth of GaN films. In addition, the weak force between the epitaxial layer and graphene can make the epitaxial layer be easily released from the substrate and transferred to other materials, realizing some novel device applications, such as flexible devices^15,16^. However, the lack of dangling bonds on graphene suppresses the GaN nucleation, limiting the growth of continuous and smooth GaN films on graphene^17^. To solve this problem, some researches have achieved the epitaxial growth of nitrides on graphene by introducing interfacial buffers or defects on graphene. For instance, Chung et al. realized the epitaxial growth of the GaN-based LED structures on graphene/sapphire substrates by using of high-density, vertically distributed ZnO nanowalls as the interfacial buffer^10^. Besides, Chen et al. reported the epitaxial growth of AlN films on graphene by nitrogen-plasma pre-treatment^18^. Although there are a few reports on the epitaxial growth of GaN on graphene, the epitaxial growth mechanism of GaN on graphene is still not clear enough, and the role of graphene in the GaN epitaxial growth process is unclear.

Here, we successfully grew a strain-relaxed GaN film on graphene/SiC substrates by metal-organic chemical vapor deposition (MOCVD). Graphene was directly prepared on SiC substrates by thermal decomposition. The nitrogen-plasma pre-treatment was performed on graphene surface, introducing the C–N-related dangling bonds, which promoted the subsequent epitaxial growth of GaN films. The scanning transmission electron microscopy (STEM) measurements reveal that part of graphene surface was etched by nitrogen-plasma pre-treatment. The growth behavior on different areas of nitrogen-plasma-treated graphene was studied, and then a growth model was proposed based on the STEM results to explain the epitaxial growth mechanism of GaN on graphene. It is worth noting that, the graphene is found to be effective to reduce the biaxial stress in GaN films. Then, we prepared InGaN/GaN multiple quantum wells (MQWs) on the strain-relaxed GaN films. The strain relaxation in GaN films leads to the improvement of indium-atom incorporation efficiency in the MQWs, resulting in the obvious red-shift of light-emitting wavelength. Our work enhances the understanding of epitaxial growth of nitrides on graphene, and provides a new way for the fabrication of nitride long wavelength GaN-based LEDs.

## Results

**Fig. 1:**
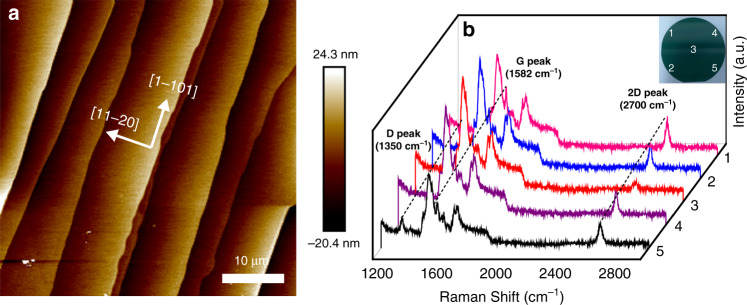
**Characterizations of graphene/SiC substrates. a** AFM image of graphene/SiC substrate, showing the step-like structure. **b** Raman spectra of five measurement positions on 2″ graphene/SiC substrate. The inset shows the photograph of as-grown 2″ graphene/SiC substrate and the measured positions

To avoid the inevitable pollution, mechanical damage, and size limitation of graphene during the transfer process, graphene was directly prepared on Si-face 4H-SiC substrates by thermal decomposition. Figure 1a shows surface morphology of the prepared graphene on SiC substrate measured by the atomic force microscopy (AFM). The AFM image presents a step-like surface morphology with a consistent direction, uniform width and height. The measured terrace width and step height are about 5–10 μm and 10–15 nm, respectively. To characterize the uniformity of the graphene on SiC substrate, we measured Raman spectra at five different positions on a 2″ diameter graphene/SiC substrate, as shown in Fig. 1b. The inset displays the photograph of the 2″ graphene/SiC substrate and the measured positions. The characteristic G peak (~1582 cm^−1^) and 2D peak (~2700 cm^−1^) of graphene can be seen in Raman spectra of five measured positions, which indicates that the prepared graphene on SiC substrate by thermal decomposition has good uniformity^19,20^. In addition, we calculated the intensity ratio of the characteristic 2D-to-G peak (*I*_2D_/*I*_G_) of graphene, and the intensity ratio of the defect-related D peak (~1350 cm^−1^) to the characteristic G peak (*I*_D_/*I*_G_) of that. The results show that the prepared graphene on SiC substrate is the multilayer one with good quality (see Fig. S1)^21^.

**Fig. 2:**
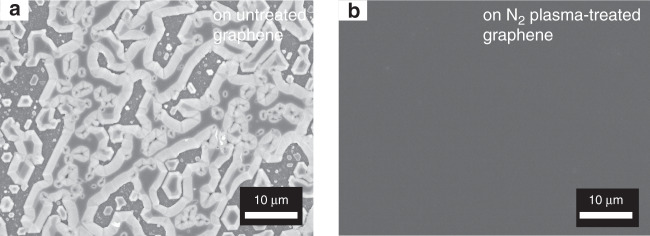
**Surface morphology of GaN on graphene/SiC.** SEM images of GaN on: **a** untreated graphene, and **b** nitrogen-plasma-treated graphene

GaN films were grown on graphene/SiC substrates using MOCVD. The lack of dangling bonds on graphene surface is not conducive to the nucleation growth of GaN^17^. The as-grown GaN on graphene cannot form a continuous film, and the surface morphology presents a discrete island-like distribution with independent nucleation, as shown in Fig. 2a. In order to enhance the nucleation ability of nitrides on graphene, we performed nitrogen-plasma pre-treatment on graphene before the epitaxial growth, and optimized the buffer layer in the epitaxial structure (see Fig. S2). Based on the above efforts, the continuous and smooth GaN films were obtained on graphene by using low- and high-temperature AlN buffers, as shown in Fig. 2b.

**Fig. 3:**
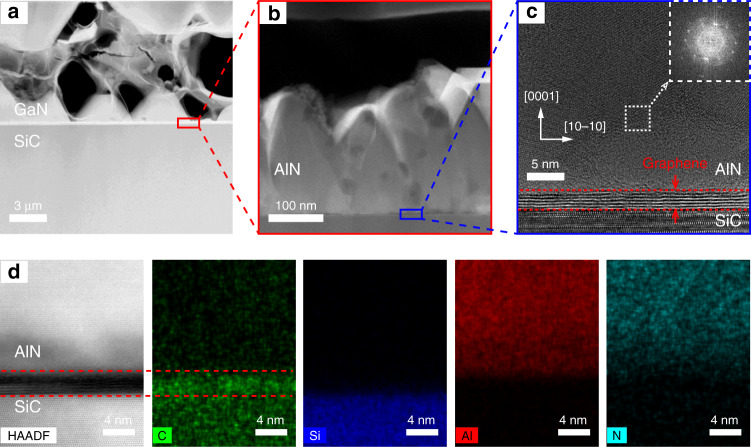
**HAADF-STEM analysis of GaN on untreated graphene/SiC. a** Cross-sectional HAADF-STEM image of GaN on untreated graphene. **b** Cross-sectional HAADF-STEM image of the interface area marked with a red rectangle frame in (**a**). **c** Cross-sectional HRTEM image of the interface area marked with a blue rectangle frame in (**b**). The multilayer graphene at the interface is clearly visible. The inset in (**c**) shows the SAED pattern of AlN in the white square region. **d** EDS mappings of elemental C, Si, Al, and N at the interface of AlN/graphene/SiC

Cross-sectional high-angle annular dark-field STEM (HAADF-STEM) measurements were performed on the as-grown GaN on untreated graphene. As shown in Fig. 3a, the epitaxial structure is composed of GaN islands, AlN buffer and graphene/SiC substrate on untreated graphene, verifying that GaN on untreated graphene shows the discrete islands growth. Figure 3b shows the HAADF-STEM image of the interface region marked with a red rectangular frame in Fig. 3a. It can be seen that the AlN buffer grown on graphene is not completely merged, and there are no GaN nucleation islands above the AlN buffer, indicating that GaN nucleation islands are distributed. Figure 3c shows the cross-sectional high-resolution transmission electron microscopy (HRTEM) image of the interface region marked with a blue rectangular frame in Fig. 3b, and the multilayer graphene at the interface can be observed clearly. The inset of Fig. 3c shows the selected area electron diffraction (SAED) pattern of the AlN buffer in the white square region in Fig. 3c. This result indicates that the AlN buffer grown on graphene is polycrystalline. Multilayer graphene can effectively screen the lattice potential field from SiC substrate, resulting in the forming of the polycrystalline AlN buffer^22^. The SEM and X-ray diffraction (XRD) measurement results also confirm that AlN buffer is polycrystalline (see Fig. S3). The interface elemental components were analyzed by energy dispersive spectroscopy (EDS) mapping. Figure 3d shows the EDS mapping images of different elements at AlN/graphene/SiC interface. The distributions of C, Si, Al, and N elements confirm that the graphene still steadily exists after AlN epitaxy, indicating that the graphene is not damaged under high temperature and hydrogen atmosphere of MOCVD system, and still maintains its integrity.

**Fig. 4:**
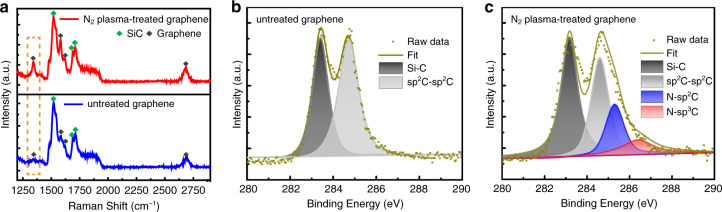
**Raman and XPS analyses of graphene before and after nitrogen-plasma pre-treatment. a** Raman spectra of untreated graphene (blue) and nitrogen-plasma-treated graphene (red). The green and black diamond blocks correspond to the characteristic peaks of SiC and graphene, respectively. The orange dashed region shows a significant increase in the intensity of D peak. XPS spectra with C 1s of: **b** untreated graphene, and **c** nitrogen-plasma-treated graphene. XPS results show that nitrogen-plasma pre-treatment introduces C–N bonds on graphene surface: N–sp^2^C bonds (~285.5 eV) and N–sp^3^C bonds (~286.5 eV)

The Raman spectra in Fig. 4a show that the D peak intensity of nitrogen-plasma-treated graphene is greatly increased compared with untreated one, which indicates that defects are introduced on graphene surface by nitrogen-plasma pre-treatment. After that treatment, the *I*_D_/*I*_G_ ratio of the graphene in Raman spectra increases from 0.3 to 0.5. The defect density in graphene can be calculated based on the *I*_D_/*I*_G_ ratio, and it increases from 9.0 × 10^10^ cm^−2^ to 2.0 × 10^11^ cm^−2^^23^. In order to further study the effect of nitrogen-plasma pre-treatment, X-ray photoelectron spectroscopy (XPS) measurements were conducted for untreated and nitrogen-plasma-treated graphene. Figure 4b shows the XPS spectrum of C 1s peak of untreated graphene, which consists of Si–C bond (~283.2 eV) from SiC substrate and sp^2^C–sp^2^C bond (~284.6 eV) from graphene^24^. After nitrogen-plasma pre-treatment, two different kinds of C–N bonding configurations appeared in C 1s peak, corresponding to N–sp^2^C bond (~285.5 eV) and N–sp^3^C bond (~286.5 eV), as shown in Fig. 4c^25^. Since the pyrrolic N atom in N–sp^3^C bond is more reactive than the pyridinic N atom in N–sp^2^C bond^11,18^, N–sp^3^C bond can provide more nucleation sites for subsequent epitaxial growth of GaN films.

**Fig. 5:**
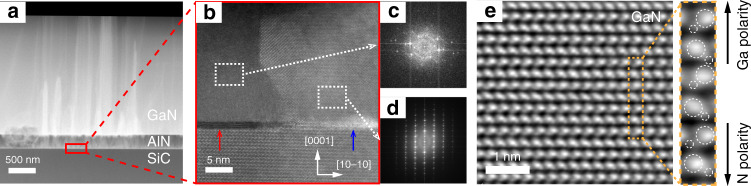
**HAADF-STEM analysis of GaN on nitrogen-plasma-treated graphene/SiC. a** Cross-sectional HAADF-STEM image of GaN on nitrogen-plasma-treated graphene. **b** Cross-sectional HAADF-STEM image of the interface area marked with a red rectangle frame in (**a**). The red arrow points to site where the dark part of the unetched graphene, whereas the blue arrow points to site where the light part of the etched graphene. **c**, **d** SAED patterns of AlN on the graphene/SiC marked with a white rectangular frame in (**b**). **e** iDPC-STEM image of GaN grown on nitrogen-plasma-treated graphene/SiC. The atomic arrangement of Ga and N atoms confirms the Ga-polarity for the as-grown GaN

Figure 5a shows the cross-sectional HAADF-STEM image of GaN on nitrogen-plasma-treated graphene. The epitaxial structure composed of GaN film, AlN buffer and graphene/SiC substrate can be observed clearly. Figure 5b shows the cross-sectional HAADF-STEM image of the interface region marked with a red rectangular frame in Fig. 5a. It is worth noting that part of graphene was etched after nitrogen-plasma pre-treatment. Among them, the dark part indicated by the red arrow is the unetched graphene region, and the light part indicated by the blue arrow is the etched graphene region. The SAED pattern in Fig. 5c indicates that the AlN buffer on the unetched graphene region is still polycrystalline, while the AlN buffer on the etched graphene region shows a single crystalline with hexagonal wurtzite structure, as shown in Fig. 5d. Single crystalline AlN plays a dominant role during the subsequent epitaxial growth, which facilitates the epitaxial growth of GaN films. Figure 5e shows the integrated differential phase contrast (iDPC) STEM image of the GaN on nitrogen-plasma-treated graphene. The arrangement of Ga and N atoms indicates that the GaN film grown on nitrogen-plasma-treated graphene is Ga polarity.

**Fig. 6:**
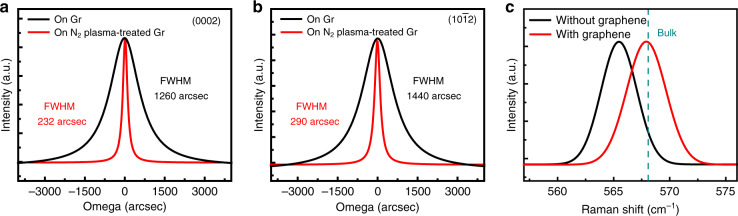
**Characterizations of GaN films on graphene/SiC.** X-ray rocking curves of: **a** (0002), and **b** (10$\bar 1$2) planes for 2 μm thick GaN films grown on untreated graphene/SiC and nitrogen-plasma-treated graphene/SiC substrates. **c** Raman spectra of GaN films epitaxially grown on graphene (red) and directly on SiC substrate (black). The green dashed line is the E_2_ (high) phonon frequency of GaN bulk material under stress-free state

To determine the macroscopic orientation and crystal structure of GaN films on nitrogen-plasma-treated graphene, we also performed XRD measurements on GaN films. The XRD 2θ and φ scan results show that GaN film on graphene is a well-aligned single crystal with hexagonal wurtzite structure (see Fig. S4). The improvement of GaN nucleation on graphene not only reduces the surface roughness of overgrown GaN, but also improves its crystalline quality, as shown in Fig. 6a, b. The full-width at half-maximum (FWHM) of (0002) and (10$\bar 1$2) planes rocking curve of GaN epilayer are greatly reduced from 1260 and 1440 arcsec to 232 and 290 arcsec, respectively. The crystalline quality of GaN films on graphene is comparable to that of GaN films grown directly on other conventional foreign substrates, such as Si and sapphire (see Table S1).

The stress in as-grown GaN films is further evaluated by Raman spectroscopy. The E_2_ (high) phonon mode in Raman spectra is sensitive to the biaxial strain of GaN films, and therefore it can be used to evaluate the biaxial stress in III-nitrides^26^. For stress-free GaN bulk materials, its E_2_ (high) phonon frequency is 568 cm^−1^^27^. Figure 6c shows that the E_2_ (high) phonon frequencies of GaN films grown on graphene and directly on SiC are both less than 568 cm^−1^, indicating that both GaN films are under tensile stress state^28^. While, the E_2_ (high) phonon frequency of GaN on nitrogen-plasma-treated graphene is closer to that of stress-free GaN, which is 567.9 cm^−1^. The specific biaxial stress value *σ* of GaN epilayers can be calculated by the formula: *σ* = *Δω/κ*, where *κ* is the stress coefficient, *Δω* is the Raman frequency shift relative to the E_2_ (high) phonon frequency of stress-free GaN films. Here, *κ* = −3.4 cm^−1^ GPa^−1^ is adopted for the calculation^29^. The calculated residual tensile stress value for the GaN films on graphene is only 0.03 GPa, which is much lower than that of GaN films grown directly on SiC substrate (0.74 GPa). This result shows that the residual stress in GaN films can be significantly reduced by inserting graphene.

**Fig. 7:**
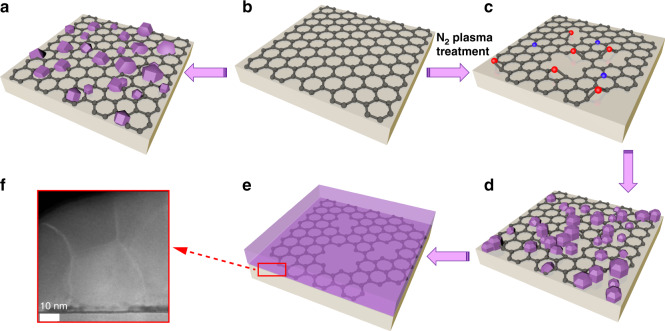
**Schematic diagram of the growth model of GaN films on nitrogen-plasma-treated graphene.** **a** AlN nucleation islands on untreated graphene. The nucleation orientation shows a random in-plane orientation. **b** Direct growth of graphene on SiC substrate. **c** As-grown graphene after nitrogen-plasma pre-treatment. The C–N-related dangling bonds were formed on nitrogen-plasma-treated graphene, and the gray, red, and blue spheres represent the C, pyrrolic N, and pyridinic N atoms, respectively. **d** AlN nucleation islands on nitrogen-plasma-treated graphene. The nucleation orientation of AlN on the etched graphene region continues the orientation of SiC substrate, which is a single crystal with *c*-axis orientation. **e** Epitaxial growth of continuous GaN films on AlN buffer. **f** Cross-sectional HAADF-STEM image of the interface at AlN/graphene/SiC

Based on the above results, we propose a growth model to explain the growth mechanism of GaN films on graphene. As shown in Fig. 7a, AlN nucleation islands grown directly on untreated graphene exhibit a random in-plane orientation. After nitrogen-plasma pre-treatment on graphene, part of its surface area is etched, as shown in Fig. 7c. At the same time, C–N-related dangling bonds are formed on graphene surface. In Fig. 7d, the orientation of AlN on etched graphene region continues the orientation of SiC substrate, showing a single crystalline *c*-axis orientation growth. The *c*-axis-oriented AlN nucleation islands gradually occupy the dominant role, and a single crystal AlN layer with the same orientation is formed by lateral epitaxy. The subsequent GaN films are grown on the AlN layer, as shown in Fig. 7e. From the cross-sectional HAADF-STEM image, we can see the grain boundaries generated during the lateral merging of AlN above different regions of graphene, as shown in Fig. 7f, which further verifies the rationality of the proposed growth model.

**Fig. 8:**
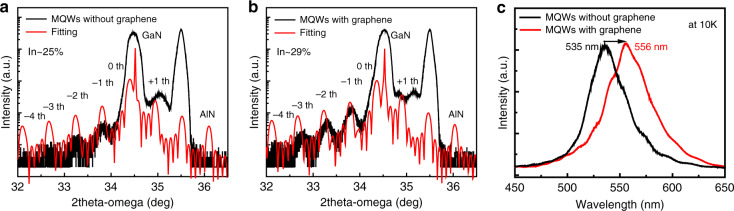
**Characterizations of InGaN/GaN MQWs on SiC and graphene/SiC substrates, respectively.** XRD 2θ-ω scans spectra of (0002) plane for InGaN/GaN MQWs grown on: **a** SiC, and **b** graphene/SiC substrates, respectively. **c** PL spectra of InGaN/GaN MQWs grown on SiC and graphene/SiC substrates (*T* = 10 K)

Based on above results, InGaN/GaN MQWs were grown on the as-grown GaN/graphene/SiC template. For comparison, we also prepared InGaN/GaN MQWs with the same structure on GaN/SiC template under the same conditions. The two samples were grown in one run. Figure 8a, b exhibit XRD 2θ scan spectra for InGaN/GaN MQWs, where intense diffraction peaks from GaN epilayer and satellite peaks from the InGaN/GaN MQWs up to the fourth order can be observed in both samples, indicating good crystalline quality and sharp interfaces of InGaN/GaN MQWs. From the fitting results, it can be found that the indium (In) content in InGaN well layer increases from 25% to 29% after inserting graphene. Figure 8c shows the PL spectra of InGaN/GaN MQWs at a low temperature of 10 K, which indicates that the insertion of graphene results in a red-shift of MQWs emission wavelength from 535 to 556 nm, and the shift is about 21 nm. Based on the peak positions of the PL spectra, the calculated In content in the InGaN/GaN MQWs on graphene/SiC is 3.5% higher than that on SiC, which is close to the XRD fitting results (In content increased by 4%). According to the Raman results, it is found that the stresses are greatly different in the GaN templates on graphene/SiC and SiC. The residual stress in the GaN template on graphene/SiC is much lower than that on SiC. Therefore, we think that the strain relaxation is the main reason for the increase of In content in InGaN/GaN MQWs.

## Discussion

In conclusion, we realized the epitaxy of strain-relaxed GaN films on graphene/SiC substrates. Graphene was directly prepared on SiC substrates by thermal decomposition. The pre-treatment of graphene with nitrogen-plasma can generate N–sp^3^C dangling bonds, which facilitate the nucleation and growth of subsequent epitaxy. STEM results provide direct evidence that the surface of graphene is partially etched by nitrogen-plasma pre-treatment. Based on the growth behavior on different etched areas of graphene surface after pre-treatment, a growth model is proposed to explain the epitaxial growth mechanism of GaN films on graphene. It is worth noting that, graphene can effectively reduce the biaxial stress in GaN films and the strain relaxation improves the incorporation efficiency of In atoms in InGaN/GaN MQWs. This work provides a feasible way for the epitaxy of strain-relaxed GaN films and opens up a new avenue for the fabrication of GaN-based long wavelength LEDs.

## Materials and methods

### Preparation of graphene

Graphene was prepared on Si-face 4H-SiC substrates by thermal decomposition. Firstly, 4H-SiC substrates were annealed in an Ar atmosphere at 800–1000 °C for 1 h to remove scratches and oxygen on the surface of SiC substrate. Then, SiC substrates was heated to 1600–1700 °C in an ultra-high purity Ar atmosphere under 800 mbar for 2–5 h to realize surface graphitization.

### Epitaxial growth of GaN films and InGaN/GaN MQWs

GaN films were grown on graphene/SiC substrates by an AIXTRON 3 × 2″ FT MOCVD system. Trimethylgallium (TMGa), trimethylaluminum (TMAl), and ammonia (NH_3_) were used as Ga, Al, and N precursors, respectively. H_2_ and N_2_ were used as carrier gases. Prior to the deposition, graphene/SiC substrates were thermally cleaned in H_2_ atmosphere at 1100 °C for 5 min. The growth began with the low-temperature AlN buffer layer of about 40 nm thickness grown at 780 °C. Then, the growth temperature ramped up to 1080 °C for the growth of high-temperature AlN buffer layer with thickness of about 160 nm. Subsequently, a 2 μm thick undoped GaN layer was grown at 1050 °C. After that, the InGaN/GaN MQWs were grown on the undoped GaN layer. The MQWs consist of 5 pairs of 2.5 nm thick InGaN quantum well (QW) layers and 15 nm thick GaN quantum barrier (QB) layers. QW and QB layers were grown at 712 and 840 °C, respectively, and they were undoped. For comparison, InGaN/GaN MQWs without graphene interlayer were fabricated directly on SiC substrate. It is worth noting that these two kinds of MQWs samples were grown in one run.

### Characterization

The surface morphology of graphene and GaN films were measured by SEM (Jeol-7500F) and AFM in tapping mode (Bruker Dimension ICON-PT). Raman spectroscopy (Renishaw inVia, 532 nm laser excitation) was performed to characterize the quality of graphene and the stress state of GaN films. The chemical states of graphene were measured by an ESCALab 250 Analytical XPS spectrometer with a monochromatic X-ray source (Al Kα, hν = 1486.6 eV). The binding energies of the spectra were referred to that of the C 1s peak at ~284.8 eV. The crystalline quality of GaN films characterized with a Rigaku Ultima IV XRD with Cu Kα radiation (0.154056 nm), and the structural properties of GaN films were investigated by aberration corrected Thermo Fisher Scientific Titan Cubed Themis G2 transmission electron microscope operated at 300 kV and equipped with Bruker Super-X EDX detector. The PL spectra of InGaN/GaN MQWs were measured by a PL system which includes a He-Cd laser (325 nm, 30 mW) as an excitation source, a Jobin Yvon iHR550 spectrometer, a Syncerity charge coupled device (CCD) and a closed circle helium cryostat.

## Supplementary information

Supplementary information for demonstration of epitaxial growth of strain-relaxed GaN films on graphene/SiC substrates for long wavelength light-emitting diodes.
