# Supplementary material for: Demonstration of epitaxial growth of strain-relaxed GaN films on graphene/SiC substrates for long wavelength light-emitting diodes
Source: Light Sci Appl. 2021 Jun 3;10:117. doi: 10.1038/s41377-021-00560-3 (PMC8175549; doi:10.1038/s41377-021-00560-3)
Supplement: Supplementary file 1 — Supplementary information for demonstration of epitaxial growth of strain-relaxed GaN films on graphene/SiC substrates for long wavelength light-emitting diodes. [file 41377_2021_560_MOESM1_ESM.docx]

**SUPPLEMENTARY INFORMATION FOR**

**Demonstration of Epitaxial Growth of Strain-Relaxed GaN Films on Graphene/SiC Substrates for Long Wavelength Light-Emitting Diodes**

Ye Yu^1^, Tao Wang^2^, Xiufang Chen^3*^, Lidong Zhang^1^, Yang Wang^1^, Yunfei Niu^1^, Jiaqi Yu^1^, Haotian Ma^1^, Xiaomeng Li^3^, Fang Liu^4^, Gaoqiang Deng^1^, Zhifeng Shi^5^, Baolin Zhang^1^, Xinqiang Wang^4^, and Yuantao Zhang^1*^

^1^State Key Laboratory of Integrated Optoelectronics, College of Electronic Science and Engineering, Jilin University, Qianjin Street 2699, Changchun 130012, China

^2^Electron Microscopy Laboratory, School of Physics, Peking University, Beijing 100871, China

^3^State Key Laboratory of Crystal Materials, Shandong University, Jinan 250100, China

^4^State Key Laboratory for Mesoscopic Physics and Frontiers Science Center for Nano-optoelectronics, School of Physics, Peking University, Beijing 100871, China

^5^Key Laboratory of Materials Physics of Ministry of Education, School of Physics and Microelectronics, Zhengzhou University, Zhengzhou 450052, China

*Correspondence: Yuantao Zhang ([zhangyt@jlu.edu.cn](mailto:zhangyt@jlu.edu.cn)) or Xiufang Chen (cxf@sdu.edu.cn)

1. **Raman characterization of graphene**

The intensity ratio of D peak to G peak (I_D_/I_G_) in Raman spectrum of graphene is usually used to calculate the defect density of graphene. The intensity ratio of 2D peak to G peak (I_2D_/I_G_) can be used to evaluate the number of layers of graphene.^[1-3]^ **Fig. S1** shows the I_D_/I_G_ and I_2D_/I_G_ ratios in Raman spectra of graphene/SiC substrates at different measured positions shown in Figure 1b. It can be seen that the I_D_/I_G_ ratios except for position 5 are about 0.3, which indicates that graphene prepared by thermal decomposition on SiC substrate is of good quality.^[4]^ The I_2D_/I_G_ ratios are lower than 1, which indicates that graphene prepared on SiC substrate is multilayer graphene.^[5]^

**

**

**Fig. S1.** The I_D_/I_G_ and I_2D_/I_G_ intensity ratios in Raman spectra of different measured positions on 2-inch graphene/SiC substrate.

1. **Growth optimization of the buffer layer structure on graphene**

We optimized the structure of buffer layer on graphene. GaN was grown on graphene by three different types of buffer layers, high-temperature GaN buffer (980 °C), high-temperature AlN buffer (1080 °C), and low-/high-temperature combined AlN buffers (780 °C/1080 °C), respectively. The SEM images of these samples are shown in **Fig. S2**. Fig. S2a shows that GaN grown on graphene with high temperature GaN buffer presents a dispersed nucleation island distribution, and the nucleation density is low. High-temperature AlN buffer layer significantly increases the nucleation density, as shown in Fig. S2b. This is because the adhesion coefficient of Al atom is larger than that of Ga atom. Al atoms are more easily adsorbed on graphene surface to form AlN buffer, which can provide more nucleation sites for subsequent GaN growth. Although AlN buffer can improve the nucleation density on graphene surface, GaN still does not form a continuous film. Furthermore, GaN films with smooth and continuous surface can be obtained by using low-/high-temperature combined AlN buffers, as shown in Fig. S2c. This is because low temperature growth can increase the nucleation density of Al atoms on graphene surface, and high temperature growth can promote the merging of adjacent AlN nucleation islands. Finally, we realized the epitaxial growth of GaN films with low-/high- temperature AlN buffers on graphene.

**
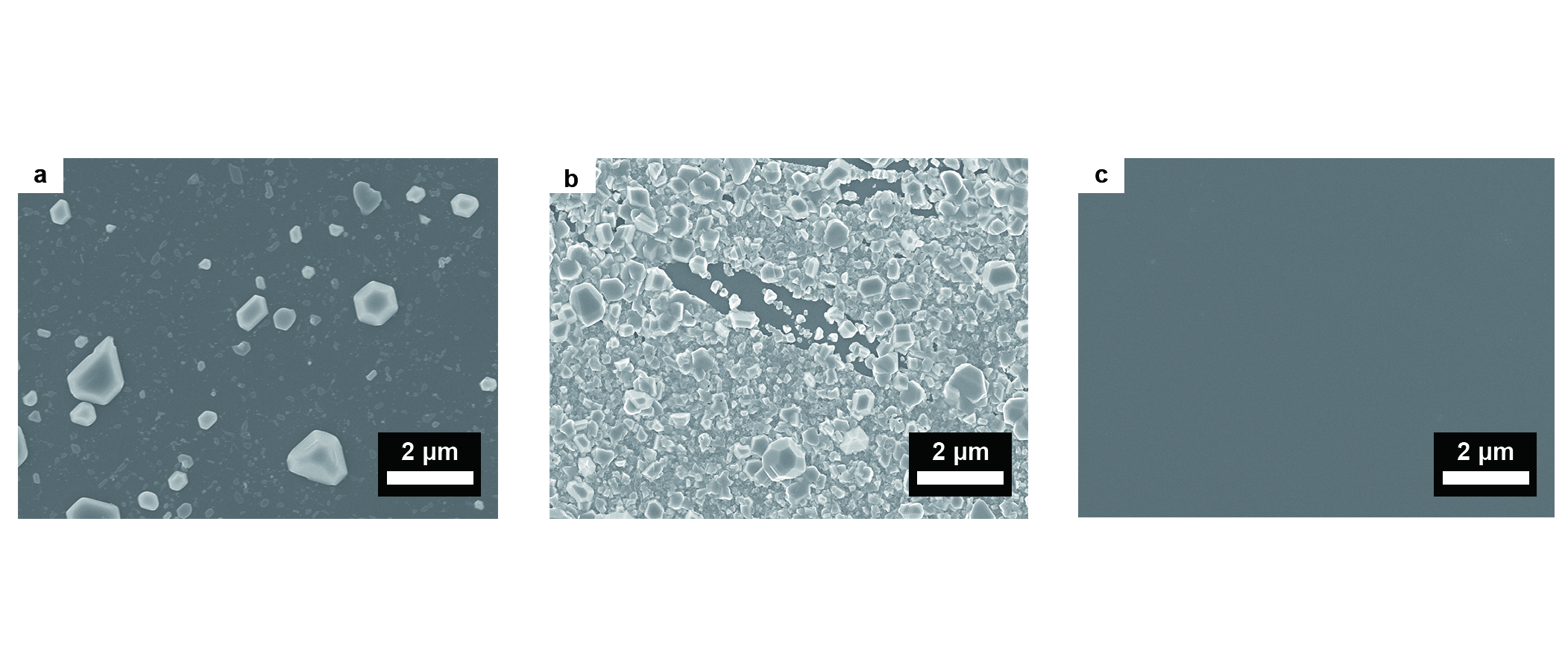
**

**Fig. S2.** SEM images of GaN grown on a) high-temperature GaN buffer (980 °C), b) high-temperature AlN buffer (1080 °C) and c) low-/high-temperature AlN buffers (780 °C/1080 °C).

1. **Epitaxial growth of AlN buffer layer on untreated graphene**

Low-/high-emperature AlN buffers were directly grown on untreated graphene. The surface morphology of the buffer is shown in **Fig. S3**a. AlN on graphene exhibits a random in-plane orientation. The XRD results show that there exist different in-plane orientations in AlN buffer, as shown in Fig. S3b.

**
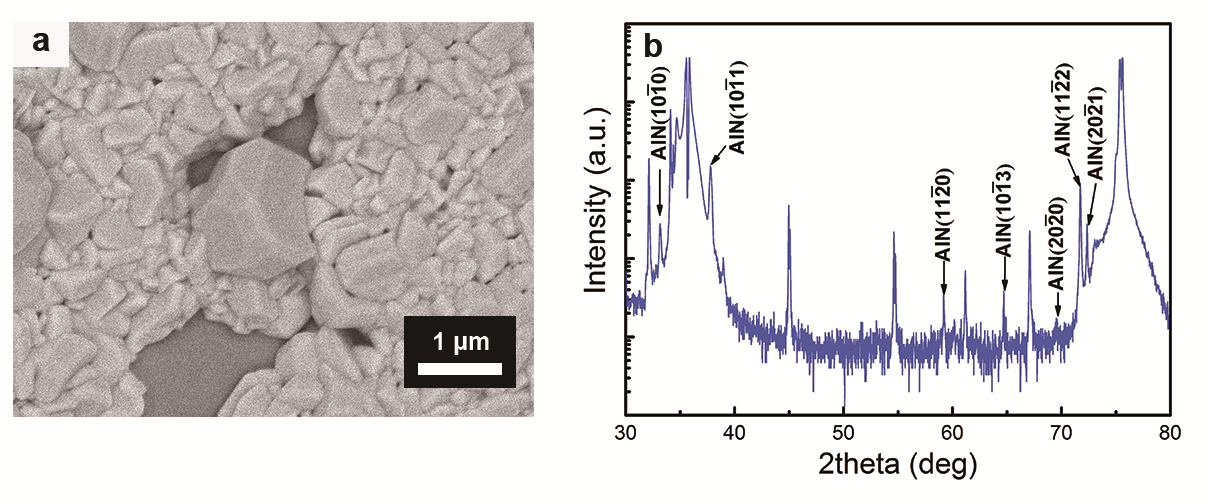
**

**Fig. S3.** Surface morphology and crystallinity of AlN buffer on untreated graphene. a) SEM image and b) XRD 2θ scan spectrum of AlN buffer on untreated graphene. The black squares correspond to the diffraction peaks of graphene/SiC substrate.

1. **XRD characterization of GaN on nitrogen-plasma-treated graphene**

To determine the macroscopic orientation and crystal structure, we performed XRD 2θ and φ scans on GaN films. As shown in **Fig. S4**a, two diffraction peaks of (0002) and (0004) planes of GaN can be seen. The diffraction peaks of GaN (10$\bar{1}$5) plane have six fold symmetry with 60° interval, as shown in Fig. S4b. The XRD 2θ and φ scan results confirm that GaN films grown on nitrogen-plasma-treated graphene are single crystalline with hexagonal wurtzite structure.

**
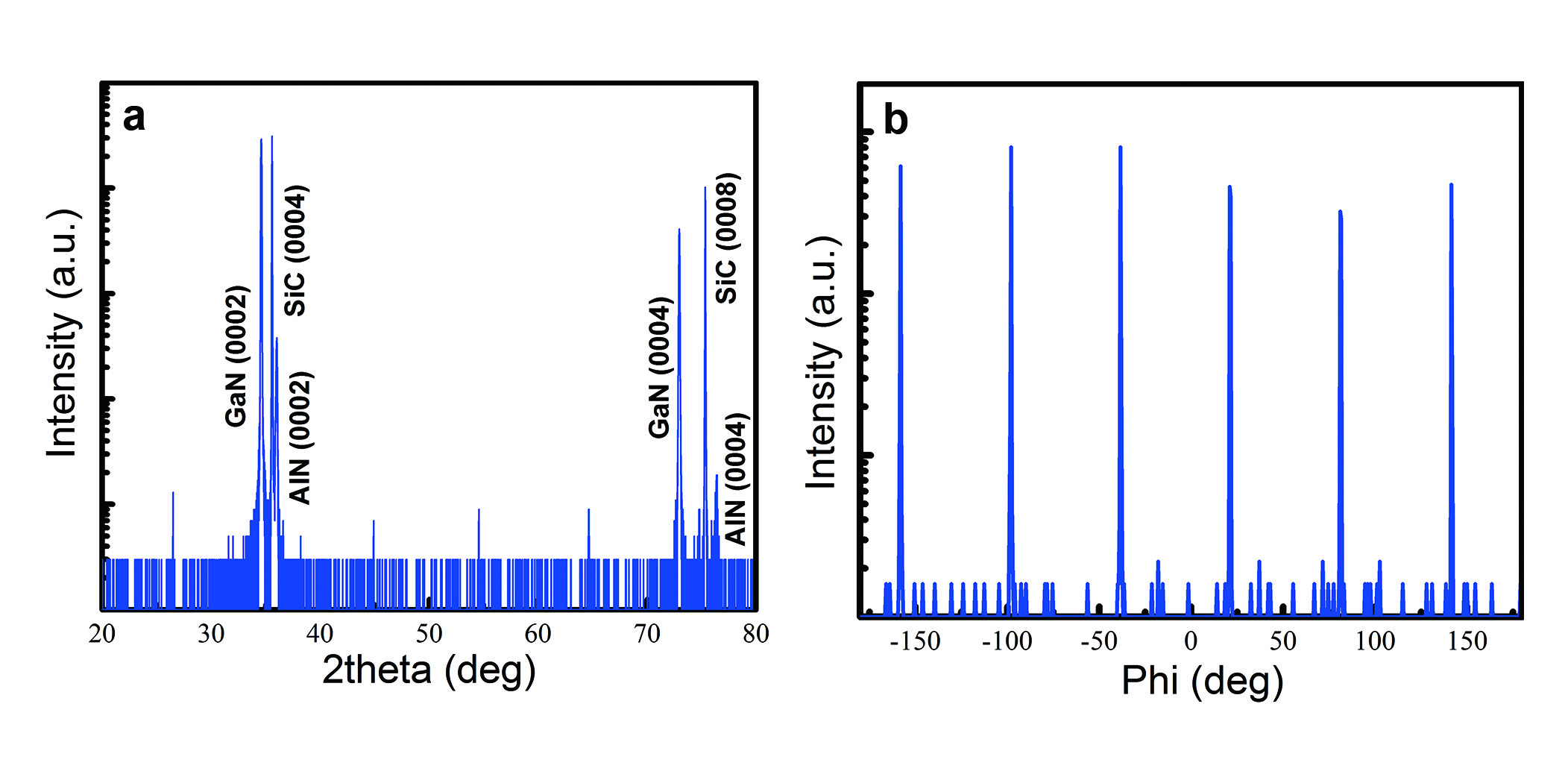
**

**Fig. S4.** a) XRD 2θ and b) φ scans spectra of GaN films on nitrogen-plasma-treated graphene.

**Table S1.** Comparison of FWHMs of X-ray rocking curves for (0002) and (10$\bar{1}$2) planes of GaN films grown on graphene and conventional foreign substrates.

|  | FWHM values (arcsec) | |
| --- | --- | --- |
|  | (0002) | (10$\bar{1}$2) |
| GaN on Gr/SiC | 232 | 290 |
| GaN on Si^[6]^ | 270 | 521 |
| GaN on Sapphire^[7]^ | 201 | 275 |

**References:**

[1] Ferrari A. C. et. al. Raman Spectrum of Graphene and Graphene Layers, *Phys. Rev. Lett.* **97**, 187401 (2006).

[2] Röhrl J. et. al. Raman spectra of epitaxial graphene on SiC(0001), *Appl. Phys. Lett.* **92**, 201918 (2008).

[3] Guo Y. et. al. The correlation of epitaxial graphene properties and morphology of SiC (0001), *J. Appl. Phys.* **115,** 043527 (2014).

[4] Yang Z. Y. et. al. A new direct growth method of graphene on Si-face of 6H-SiC by synergy of the inner and external carbon sources, *Appl. Surf. Sci.* **436**, 511-518 (2018).

[5] Kim J. et. al. Layer-Resolved Graphene Transfer via Engineered Strain Layers, *Science* **34**, 833-836 (2013).

[6] Wang K. et. al. Stress control and dislocation reduction in the initial growth of GaN on Si (111) substrates by using a thin GaN transition layer, *CrystEngComm*, **21**, 4792-4797 (2019).

[7] Chen Y. F. et. al. A study of GaN nucleation and coalescence in the initial growth stages on nanoscale patterned sapphire substrates via MOCVD, *CrystEngComm*, **20**, 6811-6820 (2018).
